# C-Reactive Protein-to-Albumin Ratio as an Early Biomarker to Identify Sepsis in Neonates with Pneumonia

**DOI:** 10.1155/2022/4711018

**Published:** 2022-07-14

**Authors:** Ping Kang, Wen Kang, Yi Li, Tiewei Li

**Affiliations:** ^1^Department of Respiratory Medicine, Children's Hospital Affiliated to Zhengzhou University, Henan Children's Hospital, Zhengzhou Children's Hospital, Zhengzhou, China; ^2^Department of Anorectal Surgery, Zhengzhou Hospital Affiliated to Jinan University, Zhengzhou, China; ^3^The Center of Henan Children's Neurodevelopmental Engineering Research, Children's Hospital Affiliated to Zhengzhou University, Henan Children's Hospital, Zhengzhou Children's Hospital, Zhengzhou, China; ^4^Zhengzhou Key Laboratory of Children's Infection and Immunity, Children's Hospital Affiliated to Zhengzhou University, Henan Children's Hospital, Zhengzhou Children's Hospital, Zhengzhou, China

## Abstract

**Objectives:**

Neonates with pneumonia often also have sepsis, and the identifying sepsis from pneumonia may be a challenge for clinicians. However, there are no available data regarding the clinical value C-reactive protein-to-albumin ratio (CAR) in identifying sepsis in neonates with pneumonia. The aim of this study was to evaluate the clinical value of CAR in identifying sepsis in neonates with pneumonia.

**Methods:**

847 neonates with pneumonia were included in this study, of which 511 neonates were diagnosed with sepsis. Neonates were divided into the sepsis group and the nonsepsis group. All neonates underwent extensive and necessary clinical and laboratory tests. CAR was calculated as serum C-reactive protein (ng/ml)/albumin (mg/ml). All statistical analyses were performed using the statistical package SPSS 24.0, as appropriate.

**Results:**

Compared with the nonsepsis group, neonates with sepsis have a higher CAR (*P* < 0.001). Further analysis showed that the prevalence of neonates with sepsis increased significantly from 41.0% in the low CAR group (CAR ≤ 0.024 × 10^−3^) to 80.0% in the high CAR group (CAR > 0.024 × 10^−3^) (*P* < 0.001). Correlation analysis showed that there was a strong positive correlation between CAR and PCT (*r* = 0.452, *P* < 0.001), nSOFA (*r* = 0.267, *P* < 0.001), and the prolonged length of hospital stay (*r* = 0.311, *P* < 0.001). Multiple logistic regression showed that CAR was an independent risk factor for the presence of sepsis in neonates with pneumonia. Receiver operating characteristic curve analysis revealed that CAR had adequate discriminatory power in predicting sepsis in neonates with pneumonia (area under curve (AUC) = 0.76, 95% CI 0.73-0.79, *P* < 0.001).

**Conclusions:**

CAR can be used as a new marker to identify sepsis in neonates with pneumonia.

## 1. Introduction

Neonatal pneumonia is a common pulmonary infectious disease in newborns caused by a variety of microorganisms, with a high mortality and morbidity. Infections acquired can occur in utero, perinatal period, or after birth[1] Neonatal sepsis is a severe infection within the bloodstream that is associated with high inflammation, hemodynamic changes, and life-threatening organ dysfunction and results in substantial morbidity and mortality[2]. According to the report by Global Sepsis Alliance (GSA), infections leading to sepsis accounted for about one-fifth of the world's neonatal deaths[3].

Neonates with pneumonia often also have sepsis, while the treatment methods of these two clinical entities are different. If neonates with pneumonia may remain unrecognized as also having the diagnosis criteria for sepsis, these patients would then not benefit from receiving early treatment of sepsis, as encouraged by the Surviving Sepsis Campaign physician's management guidelines[4]. Currently, the diagnosis of neonatal pneumonia mainly relies on radiographic examination, while the radiological features are nonspecific[5]. The gold standard for diagnosis of neonatal sepsis is blood culture[6]. However, blood culture also faces many challenges, such as a long waiting time and low positive detection rate of pathogenic microorganisms[6]. In terms of clinical symptoms, neonatal pneumonia and sepsis have some of the same clinical signs. In addition, commonly used biomarkers, such as C-reactive protein (CRP) and procalcitonin (PCT), faced variable accuracy (low sensitivity or specificity) in the prediction of neonatal sepsis[7–9]. Therefore, it is critical to identify rapid, sensitive, and specific new biomarkers to identify sepsis from pneumonia in neonates.

CRP-to-ALB ratio (CAR) is an index based on CRP and ALB level and is calculated using the formula CRP/ALB. As an emerging risk factor, CAR was closely related to multiple diseases, such as cancer, cardiovascular diseases, and sepsis[10–14]. Studies have demonstrated that sepsis can cause an increase in CRP and a decrease in ALB[15–17]. In addition, CAR has recently been considered a more useful indicator of sepsis than CRP or ALB alone in adults[15, 18]. So, we proposed that CAR may be increased in neonates with sepsis and can be a biomarker to identify neonatal sepsis. However, there are no available published data regarding the clinical value of CAR in identifying sepsis from pneumonia in neonates. Thus, this study is aimed at evaluating the clinical role of CAR in identifying sepsis from pneumonia in neonates.

## 2. Materials and Methods

### 2.1. Study Population

This is a hospital-based retrospective study conducted in Children's Hospital Affiliated to Zhengzhou University (Zhengzhou, China). 847 hospitalized neonates with pneumonia between January 2016 and December 2020 were included in this study. Inclusion criteria include (1) aged ≤28 days and (2) neonates diagnosed with pneumonia. Exclusion criteria include (1) neonates with malignancies, hematological system diseases, major congenital malformations, or other inflammatory conditions, (2) incomplete clinical and laboratory data at admission, and (3) congenital liver defects and previous liver-related diseases. The study protocol complied with the Declaration of Helsinki and obtained the approval of the hospital ethics review board.

### 2.2. Clinical Definition

The diagnosis of pneumonia was based on a combination of clinical symptoms, chest radiography, and laboratory findings. Clinical symptoms include fever, cough, respiratory distress of various degree, and hypothermia or hyperthermia. Chest radiography showed a new pulmonary infiltration. Laboratory findings usually show elevated levels of inflammatory markers. Neonatal sepsis is defined as systemic inflammatory response syndrome in the presence of or as a result of suspected or proven infection according to the published International Pediatric Sepsis Consensus[19]. The diagnosis of pneumonia and sepsis was made by two study investigators. The severity of neonatal sepsis was assessed by using the neonatal sequential organ failure assessment (nSOFA) score that consisted respiratory, cardiovascular, and hematological criteria[20].

### 2.3. Data Collection

The data of the first admission were collected from electronic medical records, including the following aspects: (1) demographic and admission status data, including age, gender, weight, body temperature, respiratory rate, heart rate, systolic blood pressure, and diastolic blood pressure; (2) laboratory data at admission, including procalcitonin (PCT), CRP, alanine aminotransferase (ALT), aspartate aminotransferase (AST), total protein (TP), and ALB. PCT concentration was measured using an Elecsys BRAHMS PCT automated electrochemiluminescence assay (B.R.A.H.M.S. Diagnostica, Hennigsdorf, Germany) on a Cobas® 8000 modular analyzer (Roche Diagnostic, Rotkreuz, Switzerland). C-reactive protein (hsCRP) was measured using a latex-enhanced immunoturbidimetric assay on an UPPER analyzer (Ultrasensitive CRP Kit, Upper Bio-Tech, Shanghai, China). ALT, AST, TP, and ALB were measured using the conventional clinical analytical method on an automatic Beckman biochemical analyzer (Beckman Coulter, California). For our data, CRP levels < 0.8 mg/l were assigned a value of 0.7 mg/l. PCT level > 100 ng/ml or <0.02 ng/ml was assigned 101 ng/ml and 0.01 ng/ml, respectively.

### 2.4. Statistical Analysis

All data statistical analyses were performed using IBM SPSS version 24.0 (SPSS Inc., Chicago, Illinois, USA). Normally distributed variables were presented as mean ± standard deviation (SD) and analyzed by independent *t*-tests. Nonnormally distributed variables were expressed as medians (interquartile range) and analyzed using the Mann–Whitney *U* test. Categorical variables are expressed as numbers (percentage) and analyzed using chi-square tests. Spearman's correlation method was used to determine the correlations between CAR and other clinical and laboratory indexes. Multiple logistic regression analysis was used to identify the independent risk factor for the presence of neonatal sepsis. Variables with a *P* value < 0.05 in the univariate logistic analysis were included in the multiple logistic regression analysis. The predictive value of CAR for the presence of neonatal sepsis was determined using the receiver operating characteristic (ROC) curve. Youden's index was calculated (sensitivity + specificity − 1) to determine the optimal cutoff point. The area under the ROC curve (AUC) of the two variables was compared using Delong's test. A two-sided *P* value of less than 0.05 was considered statistically significant.

## 3. Results

### 3.1. Study Population Characteristics

A total of 847 neonates with pneumonia were enrolled in this study. Characteristics of the study population are shown in Table 1. 511 neonates were diagnosed with sepsis (sepsis group) and the remaining 336 neonates without sepsis served as the nonsepsis group. Compared with the nonsepsis group, neonates in the sepsis group were older (7 (4.0, 13.0) vs. 11 (5.0, 17.0) days, *P* < 0.001) and had a higher body temperature (37.0 ± 0.5 vs. 37.4 ± 0.8°C, *P* < 0.001), respiratory rate (46.9 ± 8.2 vs. 50.6 ± 10.5 rate/minute, *P* < 0.001), and heart rate (142.5 ± 17.3 vs. 151.0 ± 18.4 bpm, *P* < 0.001). Biochemical analysis showed that neonates with sepsis had a higher level of PCT (0.15 (0.10, 0.25) vs. 0.34 (0.14, 1.73) ng/ml, *P* < 0.001), CRP (0.7 (0.7, 0.7) vs. 0.7 (0.7, 17.6) mg/dl, *P* < 0.001), and ALT (25.6 (20.1, 33.3) vs. 29.0 (22.4, 39.0) U/l, *P* < 0.001) and a lower level of TP (56.67 ± 7.09 vs. 53.70 ± 7.37 g/l, *P* < 0.001) and ALB (33.34 ± 4.20 vs. 30.18 ± 4.86 g/l, *P* < 0.001). Further analysis showed that neonates with sepsis had a higher CAR (0.021 (0.019, 0.024) vs. 0.028 (0.023, 0.61), *P* < 0.001) and nSOFA score (0 (0, 0) vs. 0 (0, 2.0), *P* < 0.001) and a longer length of hospital stay (9.0 (8.0, 12.0) vs. 15.0 (11.0, 23.0) days, *P* < 0.001).

### 3.2. Associations between CAR and the Presence and Severity of Neonatal Sepsis

According to the median of CAR, neonates were divided into two groups: low CAR group (≤0.024 × 10^−3^) and high CAR group (>0.024 × 10^−3^). As shown in Table 2, neonates in the high CAR group had a higher level of PCT and nSOFA score and a longer length of hospital stay. Further analysis showed that the prevalence of sepsis showed a significant increase from 41.0% in the low CAR group to 80.0% in the high CAR group, while the prevalence of pneumonia was more likely in the low CAR group (*P* < 0.001).

### 3.3. Correlation between CAR and Clinical and Laboratory Indexes

As shown in Table 3, CAR was negatively correlated with weight (*r* = −0.080, *P* = 0.021) and TP (*r* = −0.522, *P* < 0.001) and positively correlated with body temperature (*r* = 0.126, *P* < 0.001), respiratory rate (*r* = 0.164, *P* < 0.001), heart rate (*r* = 0.124, *P* < 0.001), and PCT (*r* = 0.452, *P* < 0.001). In addition, CAR also showed a positive correlation with the nSOFA score (*r* = 0.267, *P* < 0.001) and length of hospital stay (*r* = 0.311, *P* < 0.001).

### 3.4. Predictive Value of CAR for Sepsis in Neonates with Pneumonia

Variables with a *P* value < 0.05 in the univariate logistic analysis included age, temperature, heart rate, respiratory rate, CRP, AST, ALT, and TP. After adjusting these indexes, multivariable logistic regression analysis revealed that CAR was still an independent risk factor for sepsis (OR = 9.592, 95% CI 9.592-23.346, *P* < 0.001) in neonates with pneumonia. Further analysis showed that higher CAR was also independently associated with an increased prevalence of neonatal sepsis (Table 4).

### 3.5. Diagnostic Value of CAR in Neonatal Sepsis

**Figure 1:**
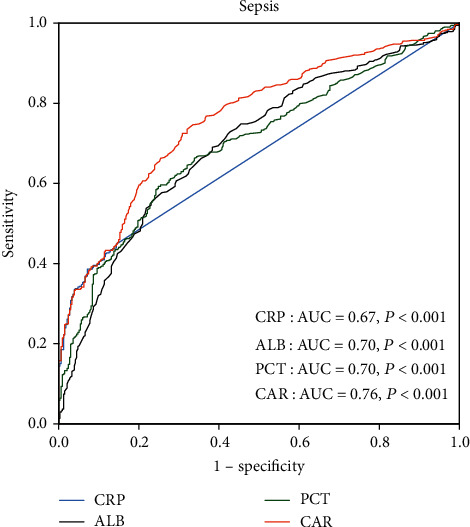
ROC curve of CRP, ALB, PCT, and CAR in predicting sepsis in neonates with pneumonia.

ROC curve analysis was performed for CAR for the prediction of sepsis in neonates with pneumonia. As shown in Figure 1, the AUC for CAR in predicting sepsis was 0.76 (95% CI 0.73-0.79, *P* < 0.001). The optimal cutoff value of CAR was 0.023 × 10^−3^, with 73% sensitivity and 69% specificity. Additionally, we also evaluated the ability of CRP and ALB to predict severe sepsis. The AUC for CRP, ALB, and PCT was 0.67 (95% CI 0.64-0.71, *P* < 0.001), 0.70 (95% CI 0.66-0.74, *P* < 0.001), and 0.70 (95% CI 0.66-0.79, *P* < 0.001), which were lower than the AUC for CAR in predicting sepsis in neonates with pneumonia (*P* < 0.05).

## 4. Discussion

Compared with adults, neonates were more susceptible to infection and then develop into pneumonia and sepsis[21]. Neonates with pneumonia may remain unrecognized as also having the diagnosis criteria for sepsis. However, the treatment methods of these two clinical entities are different. If neonates with both pneumonia and sepsis are still treated as pneumonia, they would not be benefit from the early treatment of sepsis. Currently, identifying sepsis from nonseptic neonates with pneumonia remains a challenge for clinicians using clinical criteria alone. Circulating blood biomarkers have advantages in terms of convenience, economical, and rapid, which may provide important information in identifying these two clinical entities.

Sepsis is characterized by an excessive inflammatory response to infectious pathogens and biomarker of inflammation plays an important role in predicting the presence of neonatal sepsis. CRP is an acute inflammatory protein produced by the liver and significantly increases when there is inflammation or infection in your body. Studies have demonstrated that CRP was an important predictor and risk factor for sepsis and pneumonia[22–26]. ALB was the most abundant protein found in blood produced by the liver. ALB is the most widely used clinical index of nutrition and commonly used to evaluate the nutritional status of the body[27]. Currently, studies revealed that ALB is also a marker associated with inflammation[27–34]. Hypoalbuminemia could widely be seen in patients with inflammatory diseases and was associated more severe inflammation[30, 31]. In terms of sepsis, studies reported that patients with sepsis had a lower level of ALB and low ALB level was associated with a poorer prognosis[35–37]. Compared with neonatal pneumonia, our data revealed that neonates with sepsis had a higher CRP level and a lower ALB level, indicating that CRP and ALB may have the power in identifying sepsis from nonseptic neonates with pneumonia.

CAR is determined by dividing the CRP level by the ALB level. Multiple studies have shown that CAR was valuable in predicting the prognosis of patients with certain cancers, such as lung cancer[38], colorectal cancer[39], and renal cell carcinoma[40]. In recent years, CAR has emerged as a novel marker of inflammation and has been recognized by clinicians and researchers. Studies have reported that CAR could be a reliable inflammation marker for increased coronary thrombus burden[10], acute kidney injury development[41], and sepsis[13]. In case of sepsis in neonates, Li et al.[15] reported that the CAR was an independent predictor for the presence and severity of neonatal sepsis. However, it is still unclear whether CAR can identify sepsis from pneumonia in neonates.

In this study, we firstly evaluate the clinical value of CAR in identifying sepsis from pneumonia in neonates. Our data showed that CAR was higher in neonates with sepsis and the prevalence of sepsis increased significantly from 41.0% in the low CAR group to 80.0% in the high CAR group (*P* < 0.001). Multivariate analysis showed that CAR was an independent risk factor for the presence of sepsis in neonates with pneumonia. ROC curve analysis showed that CAR had a higher discriminatory power than CRP, ALB, and PCT in predicting sepsis in neonates with pneumonia.

Our study also has several limitations. First, this is a hospital-based retrospective study, and the results further need to be confirmed by multicenter clinical studies. Second, neonatal sepsis was diagnosed based on their clinical features and was not confirmed by positive blood culture. Therefore, the accurate incidence rate of neonatal sepsis may be underestimated or overestimated. Finally, we only measured CAR at admission and believed that serial CAR measurements may be more useful in monitoring the responses to therapies and evaluating the severity of neonatal sepsis.

## 5. Conclusions

In conclusion, our study demonstrated that CAR was higher in neonates with sepsis and can be a useful early biomarker to identify sepsis in neonates with pneumonia. These results indicated that neonates with pneumonia, who also have high CAR, have a higher risk of sepsis.

## Figures and Tables

**Table 1:** Basic characteristics of study subjects by groups.

Variables	Nonsepsis group (*n* = 336)	Sepsis group (*n* = 511)	*P*
Age (days)	7.0 (4.0, 13.0)	11.0 (5.0, 17.0)	<0.001
Male, *n* (%)	197 (59.0%)	314 (61.2%)	0.517
Weight (kg)	3.3 ± 0.5	3.2 ± 0.7	0.025
Temperature (°C)	37.0 ± 0.5	37.4 ± 0.8	<0.001
Respiratory (rate/minute)	46.9 ± 8.2	50.6 ± 10.5	<0.001
Heart rate (bpm)	142.5 ± 17.3	151.0 ± 18.4	<0.001
SBP (mmHg)	76.6 ± 6.9	76.1 ± 9.0	0.365
DBP (mmHg)	46.6 ± 7.3	46.3 ± 8.7	0.594
Biochemical parameters			
PCT (ng/ml)	0.15 (0.10, 0.25)	0.34 (0.14, 1.73)	<0.001
CRP (mg/l)	0.7 (0.7, 0.7)	0.7 (0.7, 17.6)	<0.001
ALT (U/l)	25.6 (20.1, 33.3)	29.0 (22.4, 39.0)	<0.001
AST (U/l)	37.6 (30.0, 50.5)	28.3 (27.9, 54.7)	0.965
TP (g/l)	56.67 ± 7.09	53.70 ± 7.37	<0.001
ALB (g/l)	33.34 ± 4.20	30.18 ± 4.86	<0.001
CAR (×10^−3^)	0.021 (0.019, 0.024)	0.028 (0.023, 0.61)	<0.001
nSOFA	0 (0, 0)	0 (0, 2.0)	<0.001
Length of hospital stay (days)	9.0 (8.0, 12.0)	15.0 (11.0, 23.0)	<0.001

Abbreviations: SBP: systolic blood pressure; DBP: diastolic blood pressure; PCT: procalcitonin; CRP: C-reactive protein; ALT: alanine aminotransferase; AST: aspartate aminotransferase; TP: total protein; ALB: albumin; CAR: C-reactive protein-to-albumin ratio; nSOFA: neonatal sequential organ failure assessment.

**Table 2:** Clinical and demographic characteristics according to the median of CAR.

Variables	Low CAR group (≤0.024) (*n* = 422)	High CAR group (>0.024) (*n* = 425)	*P*
Age (days)	9.0 (5.0, 15.0)	9.0 (4.0, 16.0)	0.414
Male, *n* (%)	230 (54.5%)	281 (66.1%)	0.066
PCT (mg/l)	0.09 (0.15, 0.30)	0.42 (0.15, 2.11)	0.001
Clinical data			
Pneumonia, *n* (%)	249 (59.0%)	140 (20.0%)	<0.001
Sepsis, *n* (%)	173 (41.0%)	340 (80.0%)	<0.001
nSOFA	0 (0, 0)	0 (0, 1.0)	<0.001
Length of hospital stay (days)	10.0 (8.0, 14.0)	14.0 (10.0, 22.0)^a^	<0.001

Abbreviations: PCT: procalcitonin; CAR: C-reactive protein-to-albumin ratio; nSOFA: neonatal sequential organ failure assessment.

**Table 3:** Correlations between CAR and clinical parameters.

Variables	Overall population	
	*r*	*P*
Age (day)	-0.005	0.887
Weight (kg)	-0.080	0.021
Temperature (°C)	0.126	<0.001
Respiratory (rate/minute)	0.164	<0.001
Heart rate (bpm)	0.124	<0.001
PCT (ng/l)	0.452	<0.001
AST (U/l)	-0.063	0.066
ALT (U/l)	0.061	0.074
TP (g/L)	-0.522	<0.001
nSOFA	0.267	<0.001
Length of hospital stay (days)	0.311	<0.001

Abbreviations: PCT: procalcitonin; ALT: alanine aminotransferase; AST: aspartate aminotransferase; TP: total protein; CAR: C-reactive protein-to-albumin ratio; nSOFA: neonatal sequential organ failure assessment.

**Table 4:** Predictive value of CAR for sepsis in neonates with pneumonia.

Variables	Univariate		Multivariate^∗^	
	OR (95% CI)	P	OR (95% CI)	P
Presence of sepsis				
CAR	15.041 (6.212–36.417)	<0.001	9.592 (3.941–23.346)	<0.001
CAR group				
Low CAR	1		1	
High CAR	5.757 (4.236–7.824)	<0.001	4.884 (3.378–7.062)	<0.001

Note: ^∗^adjusted for age, temperature, heart rate, respiratory rate, CRP, AST, ALT, and TP. Abbreviations: CRP: C-reactive protein; ALT: alanine aminotransferase; AST: aspartate aminotransferase; TP: total protein; CAR: C-reactive protein-to-albumin ratio.

## Data Availability

The data used to support the findings of this study are available from the corresponding author upon request.

## Abbreviations

SBP: Systolic blood pressure
DBP: Diastolic blood pressure
PCT: Procalcitonin
CRP: C-reactive protein
ALT: Alanine aminotransferase
AST: Aspartate aminotransferase
TP: Total protein
ALB: Albumin
CAR: C-reactive protein-to-albumin ratio
nSOFA: Neonatal sequential organ failure assessment.
